# Sedentary (?) Occupations

**Published:** 1917-05

**Authors:** 


**A Monthly Review of Medicine and Surgery**

EDITOR AND PUBLISHER, DR. A. L. BENEDICT, 228 Summer St., corner of Elmwood Ave., Buffalo, (Address for all communications. Please make personal and telephone calls before 1 P. M.)

Third, in age, on the western continent. Independent, but supports professional organizations. News limited mainly to Western N. Y. and Penn.

Subscription $2.00 a year; $3.00 for two years in advance. Changes and errors in address or failure or duplication of delivery, should be reported immediately.

Yearly Volume 72 MAY, 1917 Number 10

### Sedentary (?) Occupations.

The custom of recording occupation in case histories, obviously is due to the bearing on hygiene, liability to exposure to weather, strains of various kinds, infections, etc. It is commonly understood that business men are of sedentary habit. Physicians commonly think of them as tied to a desk from 9 to 5, except for a hasty luncheon, as having little opportunity for recreation or the benefits of the open air. So far as our observation goes, referring of course to men in business for themselves and to those in some managerial office and without reference to clerks, this conception, however correct in the past, is so no longer. At any moment chosen at random during business hours, there is an average chance of finding the average business man in his office, about once in five times. He is seldom at his office before 10 or as late as 5. The luncheon period is rarely so short as an hour, and is often stated without apology by telephone clerks, as 11.30 to 3. This, of course, does not imply that the whole of the time is devoted to eating or rest, but it does mean that the business man is very far from being a desk-worker, and that the combination of business with entertainment involves hygienic risks quite as serious, as, though of opposite kind to, the former conception of a hurried and inadequate lunch. The business man is usually, to a large degree a traveling man, even if not literally a traveling salesman. Thus, he has both the disadvantages and the advantages incident to train riding, sudden change of climate and scene and of eating places, to irregular hours and enforced idleness, temptations due to freedom from the restraints of a methodic life in a restricted environment.

Quite aside from the fact that business duties themselves involve a radical change in the hygienic balance sheet of the business man, the modern office, in its heating, ventilation, lighting, furnishing, provision of toilet facilities and cleanliness and sanitary construction, is an antithesis to the description of earlier writers, literary or medical. There are survivals of the old stuffy or drafty office, with its uncomfortable chairs and stools, inadequate windows, flickering gas jets, gassy stove and accumulation of dirt, but they are rare except in small towns or the metropolis. The typic modern office is equipped with every practical mena of comfort, hygienic surroundings and saving of unnecessary labor.

